# Effect of an increase in Lp(a) following statin therapy on cardiovascular prognosis in secondary prevention population of coronary artery disease

**DOI:** 10.1186/s12872-022-02932-y

**Published:** 2022-11-08

**Authors:** Lijun Zhu, Yangliang Fang, Beibei Gao, Xiangbo Jin, Jiamin Zheng, Ying He, Jinyu Huang

**Affiliations:** 1 Department of Cardiology, Ningbo Municipal Medical Center LiHuili Hospital, Zhejiang, China; 2 Department of Cardiology, The Affiliated Hangzhou First People’s Hospital, Zhejiang University School of Medicine, Zhejiang, China

**Keywords:** Lipoprotein(a), Statin therapy, Coronary artery disease, Major adverse cardiovascular events

## Abstract

**Background:**

Lipoprotein (a) [Lp(a)] is an independent risk factor for coronary artery disease (CAD). Recent studies have indicated that statins tend to increase Lp(a) levels by 10–20%. However, the association of statin-mediated increases in Lp(a) levels with CAD has not been determined.

**Methods:**

This study included 488 patients with acute coronary syndrome (ACS) who underwent percutaneous coronary intervention (PCI). Lp(a) levels were measured at baseline and 1 month after statin therapy. The study endpoints were major adverse cardiovascular events (MACE). Hazard ratios for the MACE were adjusted for potential confounder using Cox regression.

**Results:**

After statin therapy, the mean level of Lp(a) increased by 19.3% from baseline. Lp(a) levels increased in 307 patients (62.9%) with a median elevation of 4.1 mg/dL. Patients with an increase in Lp(a) were at higher risk for MACE than those without an increase in Lp(a) (*p* = 0.044). Subgroup analyses revealed that a mild-to-moderate increase in Lp(a) was not associated with MACE, whereas there was a strong correlation between the highest quartile increase in Lp(a) (≥ 10.1 mg/dL) and MACE (HR = 2.29, 95%CI = 1.36–3.84, *p* = 0.002). This correlation was independent of baseline Lp(a) levels but not independent of on-statin Lp(a) levels.

**Conclusions:**

Severe increases in Lp(a) following statin therapy raise the risk of MACE, but a mild-to-moderate increase in Lp(a) may not affect the cardiovascular prognosis of CAD patients. Even if the baseline Lp(a) levels are low, it is necessary to continue testing for Lp(a) concentration at least once after statin.

**Supplementary Information:**

The online version contains supplementary material available at 10.1186/s12872-022-02932-y.

## Introduction

Statin, an HMG-CoA reductase inhibitor, is overwhelmingly effective in lowering low density lipoprotein cholesterol (LDL-C) levels and reducing atherosclerotic cardiovascular disease (ASCVD). It has been recognized as the first-line treatment for primary and secondary prevention of cardiovascular disease (CVD) [1, 2]. An estimated 145.8 million people (2.6%) worldwide are taking statins [3], as for patients with coronary artery disease (CAD), statins are recommended to almost everyone [4]. In spite of this, the fact that statin therapy cannot eliminate residual risks and treat all the lipoproteins that cause atherosclerosis is increasingly accepted [5].

Lipoprotein (a) [Lp(a)] is formed by the covalent binding of apolipoprotein A to a low density lipoprotein (LDL)-like particle [6]. It has the effect of promoting arteriosclerosis, inflammation, calcification, and thrombosis, and is an independent risk factor for ASCVD [7, 8]. Different from other lipid components, the plasma concentration of Lp(a) is mainly regulated by LPA gene which encodes for apolipoprotein(a), and is less affected by external factors [9]. Results on Lp(a) changes after statin therapy are inconsistent. Some studies show a neutral effect [10], while more of them show the effect of increasing Lp(a) [11, 12]. Several large scale meta-analyses summarize the existing research data and indicate that statins increase Lp(a) levels by more than 10% [13, 14]. This has raised concerns as to whether the use of statins increase the cardiovascular risk related to Lp(a). To date, the clinical correlation between statin-mediated increases in Lp(a) levels and CVD has not been demonstrated [15]. The current study was undertaken to investigate the effect of increases in Lp(a) levels following statin therapy on cardiovascular prognosis in a secondary prevention population of CAD.

## Patients and methods

### Study subjects

This study consecutively enrolled 618 patients with acute coronary syndrome (ACS) who underwent percutaneous coronary intervention (PCI) with drug-eluting stent implantation in the affiliated Hangzhou First People’s Hospital, Zhejiang University School of Medicine from January 1 to December 31, 2017.

**Fig. 1:**
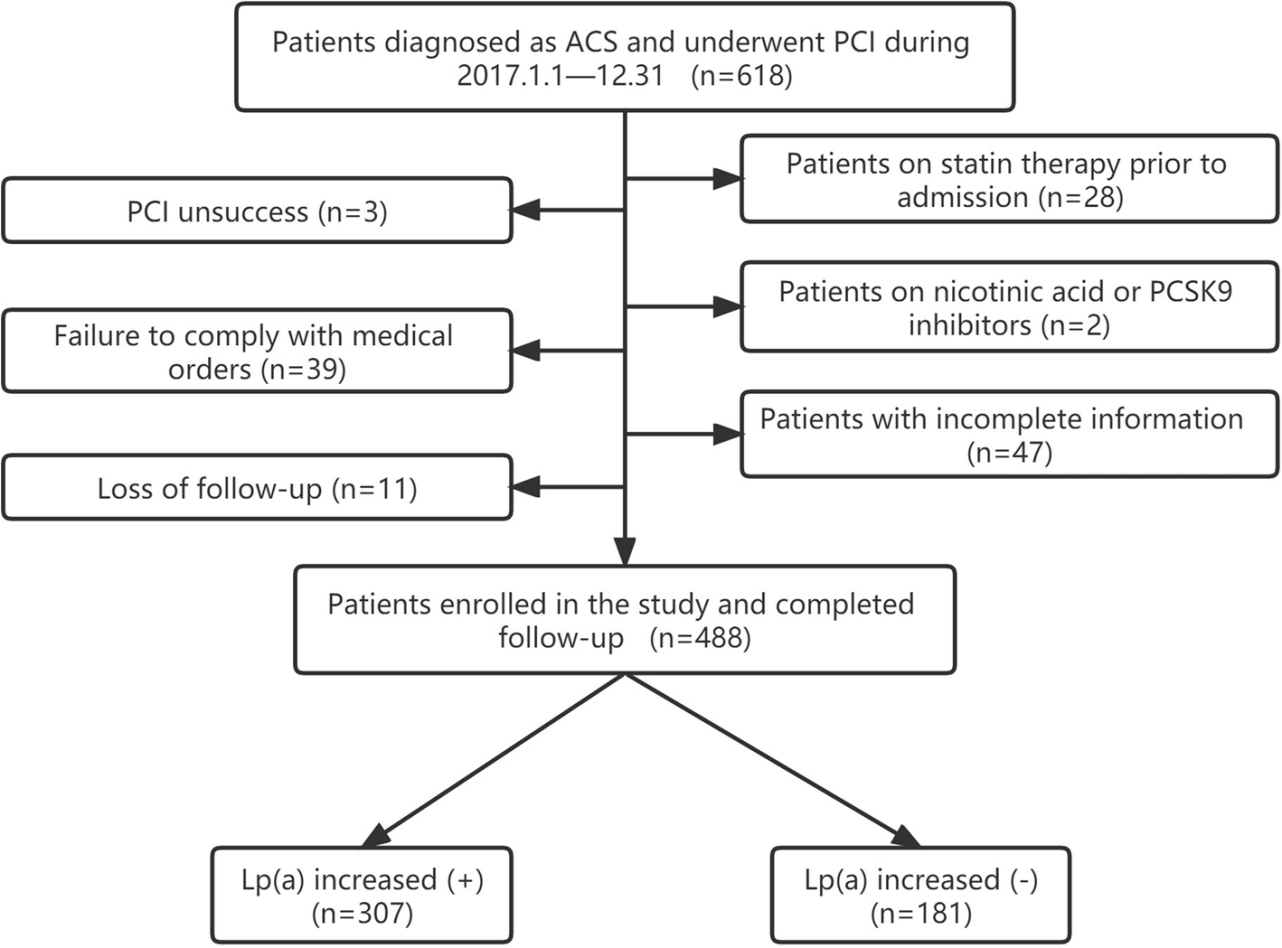
Patient flow diagram

The following subjects were excluded: 1. patients on statin therapy prior to admission (*n* = 28); 2. patients with PCI unsuccess (*n* = 3); 3. patients with incomplete basic information or follow-up data (*n* = 47); 4. patients who discontinued or changed the medication without authorization during the follow-up (*n* = 39); 5. patients on nicotinic acid or PCSK9 inhibitors (*n* = 2); 6. patients who lost follow-up (*n* = 11). As shown in Fig. 1, 488 patients finally fulfilled the inclusion criteria and were classified as the study group. All patients in the study were treated according to the the standard protocols recommended by national guidelines [16, 17].

ACS was defined as acute chest patient occurring with or without persistent ST-segment elevation as well as positive, or negative in case of unstable angina, cardiac enzymes [18]. PCI unsuccess was defined as the residual stenosis diameter of target vessel ≥ 10%, TIMI blood flow less than Grade-III, or associated in-hospital major clinical complications [19].

### Data collection

Patient characteristics were obtained from the hospital records. This included information such as age, sex, body mass index, smoking history, family history, comorbidities (hypertension, diabetes mellitus), use of secondary prevention medications (Antiplatelet agents, Lipid-lowering drugs, etc.), laboratory test results [LDL-C, Lp(a), creatinine, etc.] obtained before PCI, records of PCI and the results of blood lipids measurement after 1 month of statin therapy.

### Follow-up and study endpoints

All patients received follow-up and repeated measurement of blood lipids at Hangzhou First People’s Hospital in the first month after discharge. The Lp(a) levels were measured by the same assay methodologies. Thereafter, they were followed up at our hospital or in private care clinics every 2–3 months. Endpoint events that occurred within 3 years in patients were collected through outpatient and inpatient records. For patients without an endpoint event record at 36 months after PCI, the occurrence of events was confirmed by telephone contact.

The study endpoints were major adverse cardiovascular events (MACE), including cardiovascular death, non-fatal myocardial infarction or ischemic stroke, hospitalization related to unstable angina and unplanned coronary revascularization.

### Statistical analysis

Categorical variables were represented as number (%) and analyzed by chi-square test or Fisher’s exact test. Continuous variables that met the normal distribution were represented as mean ± S.D. and analyzed by t-test or variance analysis. Continuous variables without a normal distribution were represented as median (25th percentile,75th percentile) and analyzed by nonparametric test.

Lp(a) was converted into two variables [Lp(a) ≥ 50 mg/dL and log10-transformed Lp(a)]. The hazard ratio (HR) and 95% confidence interval (CI) for MACE were calculated respectively by Cox proportional-hazard regression models, which were used to compare the correlation of pre-and-post statin Lp(a) levels with MACE.

The cumulative MACE—free survival rates between Lp(a) increased ( +) group and Lp(a) increased (-) group were estimated via the Kaplan–Meier method, and the differences were analyzed by the log-rank test. The HR and 95%CI for MACE for each subgroup based on the quartile of the increase in Lp(a) were calculated via Cox proportional hazard models. Statistical analysis was performed using IBM SPSS Statistics, Version 25.0. (Armonk, NY), with a *p* value of < 0.05 considered significant.

## Results

### Effect of statins on Lp(a) levels

In this study, 457 (93.65%) patients had a lipid-lowering regimen consisting of statins alone, and 31 (6.35%) received statin plus ezetimibe. As shown in Table 1, the mean level of Lp(a) increased by approximately 19.3% in patients after LLT, a statistically significant difference (*p* = 0.036). Considering the possible effect of ezetimibe on Lp(a) [20], the changes of Lp(a) levels in patients taking ezetimibe and those not taking ezetimibe were compared. It showed that there was no significant difference between them (19.56% vs 19.30%, *p* = 0.972), so the influence of ezetimibe was ignored in subsequent steps.

**Table 1:** Lp(a) levels at baseline and 1-month after LLT

Lipid-lowering strategy	Baseline (mg/dL)	On-statin (mg/dL)	percent change
statin (*n* = 457)	13.0 (8.0, 28.0)	15.1 (8.3, 35.4)	+19.28%
statin + ezetimibe (*n* = 31)	14.0 (9.0, 22.0)	14.4 (9.0, 28.0)	+19.56%
Total (*n* = 488)	13.0 (8.0, 27.0)	15.1 (8.3, 35.1)	+19.30%

On-statin: after 1 month of statin therapy

Table S1 describes the detailed use of statins. There was no obvious difference in the type and dose of statins used between the two groups.

### Patient characteristics

The average age of patients was 65.9 ± 9.7 years, including 328 males (67.2%). After statin therapy, Lp(a) levels increased in 307 patients (62.9%) with a median elevation of 4.1 mg/dL, and 181 patients (37.1%) had no increase in Lp(a). Characteristics of the population are shown in Table 2. The number of diabetic mellitus patients in Lp(a) increased ( +) group was lower than that in Lp(a) increased (-) group (26.97% vs 38.67%, *p* = 0.049). The baseline Lp(a) levels were similar between the two groups [14.0 (8.0–29.0) vs 12.0 (7.0–22.0), *p* = 0.176], but significant differences were observed after statin therapy [20.7 (11.0–40.7) vs 10.0 (6.4–18.2), *p* < 0.001]. For other clinical features, there was no significant difference between the two groups (*p* > 0.05).

**Table 2:** Baseline characteristics

	Lp(a) increased ( +) (*n* = 307)	Lp(a) increased (-) (*n* = 181)	*p* value
Age[years]	66.54 ± 9.49	64.85 ± 10.50	0.067
Male [cases (%)]	202 (65.80)	126 (69.61)	0.386
BMI (kg/m^2^)	24.08 ± 3.11	24.62 ± 3.12	0.067
Diabetes mellitus [cases (%)]	92 (26.97)	70 (38.67)	0.049
Hypertension [cases (%)]	229 (74.59)	137 (75.69)	0.787
Smoking history [cases (%)]	141 (45.93)	84 (46.41)	0.918
Family history [cases (%)]	28 (9.12)	12 (6.63)	0.333
Creatinine (μmoI/L)	87.91 ± 19.00	87.30 ± 17.12	0.726
hs-CRP (mg/L)	3.0 (1.0 – 25.0)	3.0 (1.0 – 5.0)	0.491
**Angiography data**			
Single-vessel lesion^a^ [cases (%)]	100 (32.57)	48 (26.52)	0.160
Double-vessel lesions [cases (%)]	79 (25.73)	55 (30.39)	0.266
Triple-vessel lesions [cases (%)]	127 (41.37)	78 (43.09)	0.709
Left main lesion [cases (%)]	27 (8.79)	21 (11.60)	0.314
CTO [cases (%)]	61 (19.87)	37 (20.44)	0.879
Coronary stents (number)	2.42 ± 1.53	2.53 ± 1.68	0.472
**Baseline blood lipids**			0.176
TC (mmol/L)	4.43 ± 1.67	4.45 ± 1.03	0.849
LDL-C (mmol/L)	2.57 ± 0.96	2.59 ± 0.82	0.820
HDL-C (mmol/L)	1.09 ± 0.27	1.08 ± 0.28	0.759
Lp(a) mg/dL	14.0 (8.0 – 29.0)	12.0 (7.0 – 22.0)	0.176
TG (mmol/L)	1.68 ± 1.41	1.68 ± 1.00	0.999
**On-statin blood lipids**			
TC (mmol/L)	3.33 ± 0.73	3.31 ± 0.81	0.796
LDL-C (mmol/L)	1.65 ± 0.57	1.67 ± 0.50	0.654
HDL-C (mmol/L)	1.13 ± 1.00	1.01 ± 0.25	0.161
Lp(a) mg/dL	20.7 (11.0 – 40.7)	10.0 (6.4 – 18.2)	< 0.001
TG (mmol/L)	1.31 ± 0.76	1.46 ± 0.78	0.031
**Medicine use**			
Statin [cases (%)]	307 (100.0)	181 (100.0)	—
Ezetimibe [cases (%)]	16 (5.21)	15 (8.29)	0.178
β blocker [cases (%)]	250 (81.43)	151 (83.43)	0.579
ACEI/ARB [cases (%)]	221 (71.99)	139 (76.80)	0.243
Antiplatelet agent (at discharge)			
DAPT [cases (%)]	307 (100.0)	181 (100.0)	—
Aspirin [cases (%)]	307 (100.0)	181 (100.0)	—
Clopidogrel [cases (%)]	242 (78.83)	136 (75.14)	0.346
Ticagrelor [cases (%)]	65 (21.17)	45 (28.86)	0.346
Antiplatelet agent (at the end of FUP)			
DAPT [cases (%)]	30 (9.77)	12 (6.63)	0.232
SAPT [cases (%)]	277 (90.23)	169 (93.37)	0.232
Aspirin [cases (%)]	268 (87.30)	162 (89.50)	0.467
Clopidogrel [cases (%)]	61 (19.87)	28 (15.47)	0.224
Ticagrelor [cases (%)]	8 (2.61)	3 (1.66)	0.495

Lp(a) increased ( +): patients with on-statin Lp(a) levels higher than baseline Lp(a) levels

Lp(a) increased (-): patients with on-statin Lp(a) levels less than or equal to baseline Lp(a) levels

*BMI* body mass index, *hs-CRP* high sensitivity C-reactive protein, *TC* total cholesterol, *LDL-C* low density lipoprotein cholesterol, *HDL-C* high density lipoprotein cholesterol, *TG* triglycerides, *Lp(a)* Lipoprotein (a), *ACEI* angiotensin converting enzyme inhibitor, *ARB* angiotensin-receptor blocker, *DAPT* dual antiplatelet therapy, *SAPT* single antiplatelet therapy, *FUP* follow up, *CTO* total coronary occlusion

^a^Lesion was defined as coronary artery diameter stenosis of more than 50%

### Association of pre-and-post statin Lp(a) levels with MACE

**Fig. 2:**
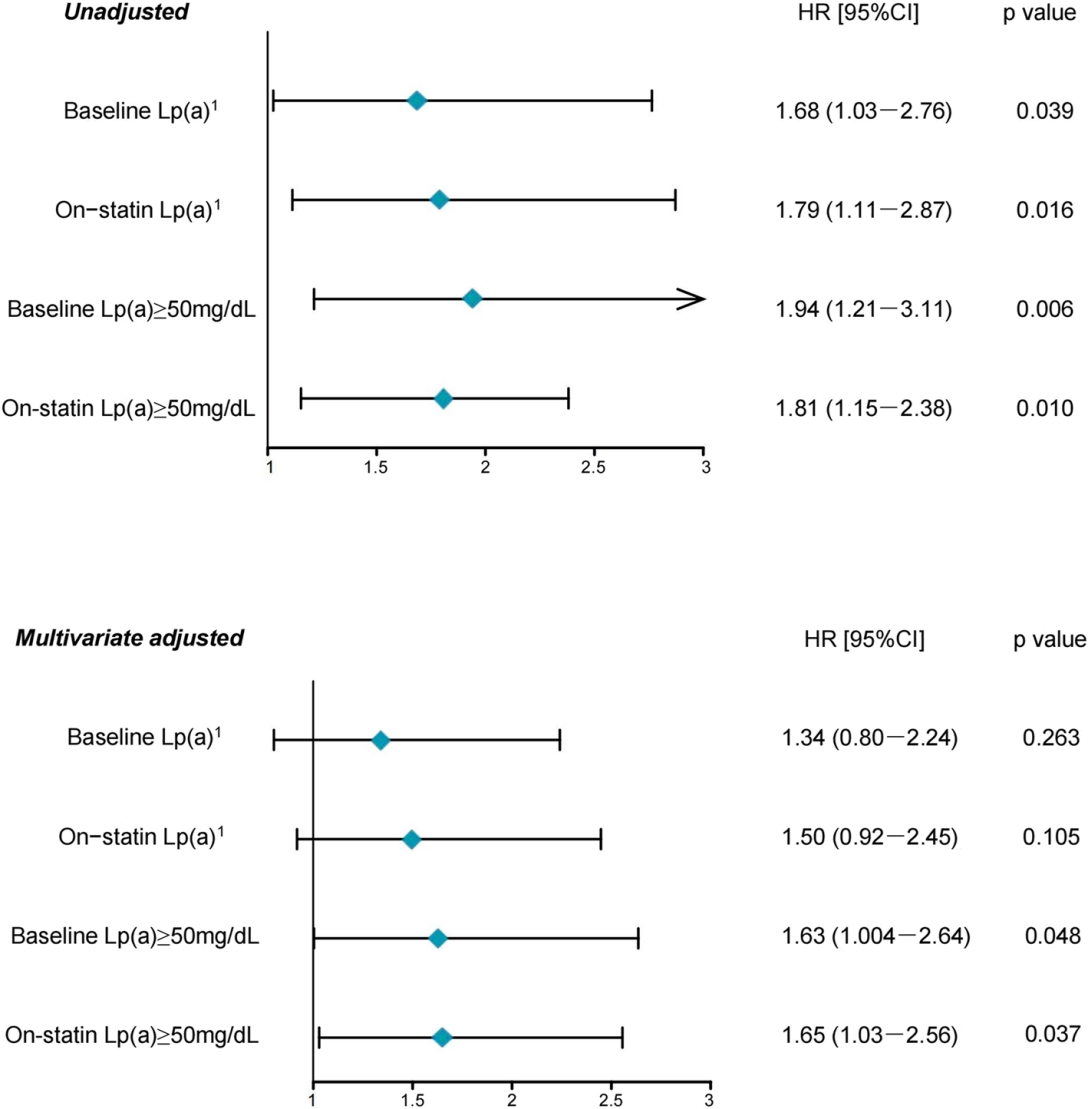
Association of baseline and on-statin Lp(a) with MACE

A total of 105 MACE (detailed in Table S2) were recorded during the 3-year follow-up period (average 31.4months). As shown in Figure 2, the association of pre-and-post statin Lp(a) levels with MACE remained unchanged substantially. Lp(a) levels ≥ 50mg/dL was an independent risk factor for MACE [Baseline: HR = 1.63, 95%CI =1.004-2.64, *p* = 0.048; On-statin: HR = 1.65, 95%CI =1.03-2.56, *p* = 0.037]. The log10-transformed Lp(a) was also associated with MACE [Baseline: HR = 1.68, 95%CI =1.03-2.76, *p* = 0.039; On-statin: HR = 1.79, 95%CI =1.11-2.87, *p* = 0.016], but the correlation became less significant after multi-factor adjustment [Baseline: HR = 1.34, 95%CI =0.80-2.24, *p* = 0.263; On-statin: HR = 1.50, 95%CI =0.92-2.45, *p* = 0.105].

Other risk factors for MACE are listed in Table 3. LDL-C and diabetes mellitus had a strong correlation with MACE, while age and hs-CRP were weak correlation factors for MACE.

**Table 3:** Risk factors for MACE

	Univariate analysis		Multivariate analysis	
	HR (95%CI)	*p*	HR (95%CI)	*p*
Age	1.02 (1.003–1.05)	0.023	1.02 (1.002–1.05)	0.033
hs-CRP	1.03 (1.008–1.04)	0.003	1.02 (1.003–1.04)	0.020
On-statin LDL-C	1.47 (1.07–2.21)	0.018	1.45 (1.05–2.01)	0.024
Diabetes mellitus	1.73 (1.18–2.54)	0.005	1.69 (1.15–2.49)	0.008

*hs-CRP* high sensitivity C-reactive protein, *LDL-C* low density lipoprotein cholesterol

In Cox proportional risk model, Lp(a) was converted into continuous variable “Lp(a)^1^” and categorical variable “Lp(a) levels ≥ 50 mg/dL”. “Lp(a)^1”^ = The log10-transformed Lp(a) level.

“Lp(a) ≥ 50 mg/dL” is generally considered a risk factor for CVD. In this study, 64 patients had baseline Lp(a) ≥ 50 mg/dL and 78 patients had on-statin Lp(a) ≥ 50 mg/dL.

Multivariate adjusted: The variables with *p* < 0.05 in univariate analysis were adjusted, including age, diabetes, LDL-C and hs-CRP. Univariate analysis is detailed in Table S3.

### Effect of statin-mediated increases in Lp(a) levels on cardiovascular prognosis

**Fig. 3:**
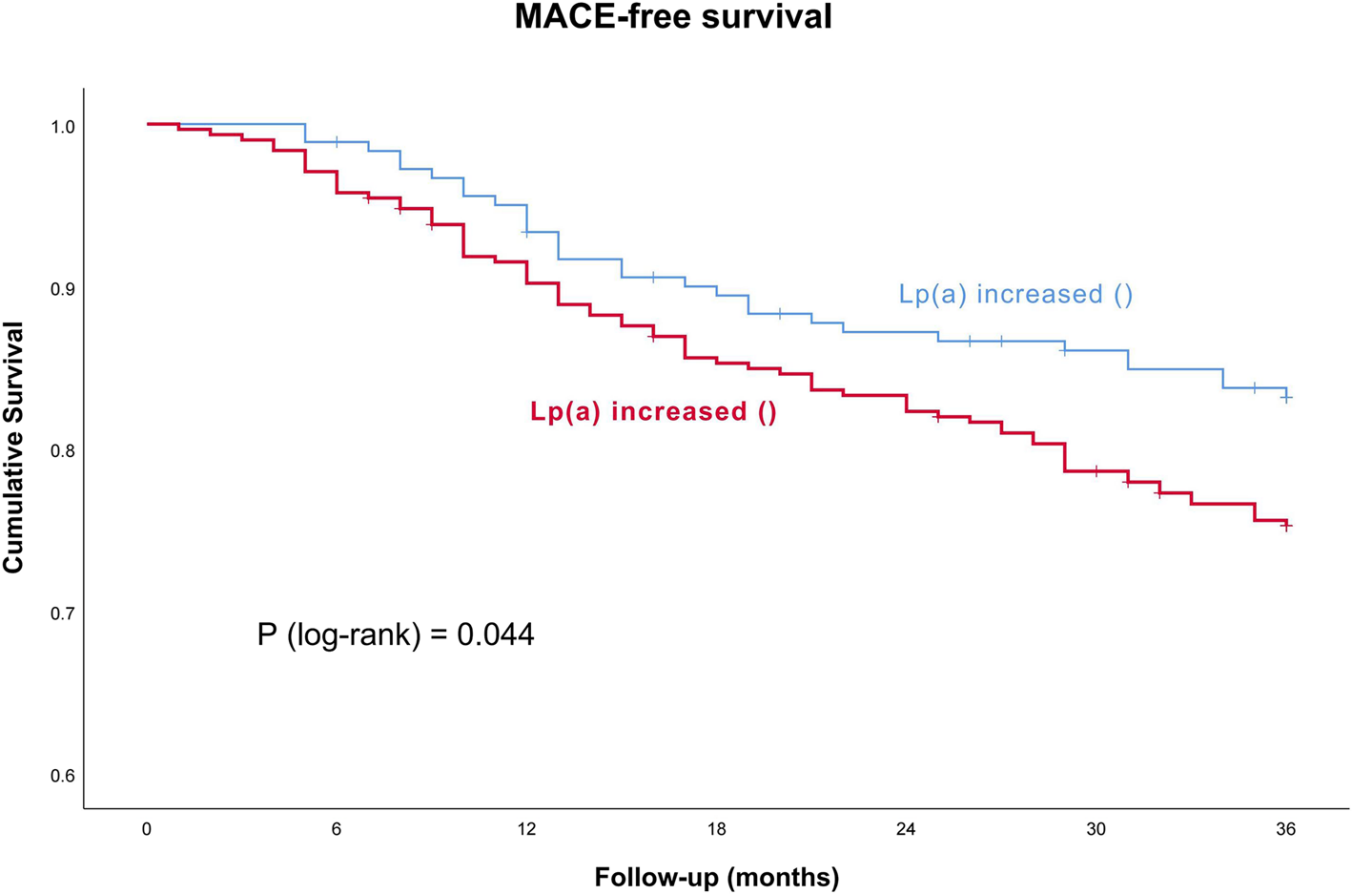
Kaplan–Meier curve model for MACE. MACE = major adverse cardiovascular events

Of the 105 patients with MACE, 75(24.43%) were in the Lp(a) increased ( +) group and 30 (16.57%) were in the Lp(a) increased (-) group. Patients with an increase in Lp(a) had a higher incidence of MACE ( *p* = 0.044, Figs. 3).

Then, patients with an increase in Lp(a) were divided into four groups based on the quartile of increases in Lp(a) levels. As shown in Table 4, patients in the highest quartile group had a significantly higher risk of MACE than patients without an increase in Lp(a) (HR = 2.29, 95CI = 1.36–3.84, *p* = 0.002). And there was no significant difference in MACE risk between other quartile groups and reference patients. (*p* > 0.05). After adjusting for baseline Lp(a) levels, the correlation did not change notablely. The highest quartile increase in Lp(a) is still closely related to MACE (HR = 2.00, 95CI = 1.18–3.42, *p* = 0.011). But this correlation disappeared in the model that adjusted for on-statin Lp(a) levels.

**Table 4:** Correlation between magnitude of increase in Lp(a) levels and MACE

	Crude model		Adjusted model I		Adjusted model II	
	HR (95%CI)	*p*	HR (95%CI)	*p*	HR (95%CI)	*p*
Reference category (*n* = 181) ≤ 0 mg/dL	1	—	1	—	1	—
1st quartile (*n* = 90) > 0-2 mg/dL	1.57 (0.90–2.71)	0.111	1.72 (0.98–3.02)	0.058	1.63 (0.94–2.83)	0.084
2nd quartile (*n* = 64) > 2–4.1 mg/dL	1.59 (0.87–2.92)	0.134	1.65 (0.90–3.02)	0.109	1.56 (0.85–2.86)	0.151
3rd quartile (*n* = 77) > 4.1–10.1 mg/dL	0.78 (0.38–1.59)	0.488	0.78 (0.38–1.59)	0.485	0.73 (0.35–1.49)	0.381
4th quartile (*n* = 76) > 10.1 mg/dL	2.29 (1.36–3.84)	0.002	2.00 (1.18–3.42)	0.011	1.78 (0.97–3.28)	0.062

Reference category: patients with an increase in Lp(a)

1st quartile: patients with an increase in Lp(a) level > 0-2 mg/dL

2nd quartile: > 2–4.1 mg/dL. 3rd quartile: > 4.1–10.1 mg/dL.4th quartile: > 10.1 mg/dL

Adjusted model I: adjusted for baseline Lp(a) levels

Adjusted model II:adjusted for on-statin Lp(a) levels

## Discussion

This study has three major findings: 1) Statin therapy increases Lp(a) levels. The mean level of Lp(a) increased by approximately 19.3% in subjects after statin. 2) Lp(a) levels were associated with MACE, and hazard ratios for Lp(a) levels at both baseline and on-statin were comparable. 3) Patients with a severe increase in Lp(a) after statin therapy have a higher risk of MACE than those without an increase in Lp(a).

Lp(a) is an emerging cardiovascular risk factor. Numerous studies have confirmed its correlation with the onset of coronary heart disease and the recurrence of adverse cardiovascular events. Emerging Risk Factors Collaboration reported that there are continuous, independent, and modest associations of Lp(a) concentration with risk of CAD and stroke. For per 3.5-fold higher than normal Lp(a) concentration, the risk of CAD increased by 13% [21]. Genome-wide association and Mendelian randomized trials showed that the variations at some loci of LPA gene were strongly associated with both an increased level of Lp(a) and an increased risk of CAD, proving the relationship between Lp(a) and CAD at the gene level [22–24]. Lp(a) accelerates the progression of low-attenuation coronary plaques (necrotic cores) [25]. As for patients with established cardiovascular disease, especially in young individuals, higher levels of Lp(a) are associated with an increased risk of MACE such as CAD death, myocardial infarction, and urgent revascularization [26–28].

Since Lp(a) level is mainly regulated by LPA gene and tends to be constant throughout life, measurement of Lp(a) after statin therapy is rarely performed in clinical practice, leading to a lack of recognition of statin-mediated increases in Lp(a) levels. However, adverse effects of statins on Lp(a) levels have been repeatedly observed in clinical trials. An ILLUMINATE trial showed that Lp(a) levels are positively and dose-dependently correlated with atorvastatin dosage [9]. De Boer et al. reported that statins are associated with approximately 1.1 mg/dL increases in Lp(a) levels compared to placebo, whereas high-intensity statins are associated with 2.6 mg/L increases in Lp(a) levels [29]. Several systematic reviews and meta-analyses most recently indicated that statins increase Lp(a) levels by 10–20% [13, 14]. In this study, statins increased the mean Lp(a) level by 19.6%, similar in extent to the current findings. The underlying mechanisms of statin-mediated increase in Lp(a) levels are not fully defined. It may be due to statin enhence LPA mRNA and apolipoprotein (a) synthesis and secretion. In addition, the increase in plasma PCSK9 protein after statin treatment may also be responsible for the increase in Lp(a) [14].

Do statin-mediated increases in Lp(a) levels cause additional cardiovascular risk? In a meta-analysis, baseline and on-statin Lp(a) levels ≥ 50 mg/dL are associated with a 1.35—and 1.42-fold increased risk of CVD, hazard ratios for high Lp(a) levels at both baseline and on statin are comparable [30]. The JUPITER trial aslo indicated that statin therapy does not significantly increase Lp(a)-associated CVD risk (HR: baseline vs On-statin = 1.18 vs. 1.27) [11]. And we come to the same result (shown in Fig. 2). However, previous studies only compared the overall association of pre-and-post statin Lp(a) levels with CVD/MACE in groups. This research is the first to explore the correlation between the increase of individual Lp(a) level and CVD/MACE after statin therapy, and proves that a significant increase in Lp(a) would raise the risk of MACE. In addition, this correlation is independent of baseline Lp(a), but dependent of on-statin Lp(a), suggesting the importance of repeated measurement of Lp(a) after statin therapy.

Based on the results, we consider it necessary to continue testing for Lp(a) levels at least once after statin therapy, even if the baseline Lp(a) levels are low. For patients with on-statin Lp(a) levels ≥ 50 mg/dL, PCSK9 inhibitors may be used as appropriate to reduce the residual cardiovascular risk [26, 31]. Several Lp(a) targeted therapies have entered into Phase II/III clinical trials and are believed to provide additional benefits to CVD patients in the near future [32–34]. However, statin use should not be hindered by fear of an increase in Lp(a). Because most patients have no or mild increase in Lp(a) after statin therapy, which does not increase the risk of cardiovascular events. Moreover, Statins play an irreplaceable role in reducing LDL-C, the chief culprit of atherosclerosis [35, 36]. Reducing LDL-C by 38.67 mg/dL results in cardiovascular benefits comparable to reducing Lp(a) by 67.5 mg/dL [37].

### Limitations

First, long-term outpatient follow-up were not all conducted in our hospital, some patients were followed up in private care clinics. We were unable to obtain accurate lipid measurement results and other clinical details for each patient after long-term statin therapy. Therefore, the prognostic implications of subsequent changes in Lp(a) levels cannot be determined. Second, the potential role of ezetimibe in the study is not analyzed. Third, this is a single-center study with a small sample size, and the results are susceptible to incidental factors. We emphasize that these results need to be verified in large-scale clinical studies.

## Conclusions

Statins increase Lp(a) levels in some patients with CAD. Severe increases in Lp(a) following statin therapy raise the risk of MACE, whereas a mild-to-moderate increase in Lp(a) may not affect the cardiovascular prognosis of CAD patients. Statin use should not be hindered for fear of an increase in Lp(a), but it is necessary to continue testing for Lp(a) concentration at least once after statin therapy.

## Supplementary Information

**Additional file 1:** **Table S1.** Statins used in study subjects. **Table S2.** Endpoint events for study subjects. **Table S3.** Univariate COX analysis of risk factors for MACE.

## Data Availability

The datasets used and analyzed during the current study are available from the corresponding author on reasonable request.

## Abbreviations

Lp(a): Lipoprotein (a)
CAD: Coronary artery disease
ACS: Acute coronary syndrome
PCI: Percutaneous coronary intervention
MACE: Major adverse cardiovascular events
LDL-C: Low density lipoprotein cholesterol
ASCVD: Atherosclerotic cardiovascular disease
CVD: Cardiovascular disease
LDL: Low density lipoprotein
ACS: Acute coronary syndrome
LLT: Lipid-lowering therapy
HR: Hazard ratio
CI: Confidence interval
BMI: Body mass index
hs-CRP: High sensitivity C-reactive protein
TC: Total cholesterol
HDL-C: High density lipoprotein cholesterol
TG: Triglycerides
ACEI: Angiotensin converting enzyme inhibitor
ARB: Angiotensin-receptor blocker
DAPT: Dual antiplatelet therapy
SAPT: Single antiplatelet therapy
FUP: Follow up
CTO: Total coronary occlusion
UA: Unstable angina
