# Correction: Effect of an increase in Lp(a) following statin therapy on cardiovascular prognosis in secondary prevention population of coronary artery disease

**DOI:** 10.1186/s12872-022-03027-4

**Published:** 2022-12-30

**Authors:** Lijun Zhu, Yangliang Fang, Beibei Gao, Xiangbo Jin, Jiamin Zheng, Ying He, Jinyu Huang

**Affiliations:** 1 Department of Cardiology, Ningbo Municipal Medical Center LiHuili Hospital, Zhejiang, China; 2 Department of Cardiology, The Affiliated Hangzhou First People’s Hospital, Zhejiang University School of Medicine, Zhejiang, China

**Correction to: BMC Cardiovascular Disorders (2022) 22:474** 10.1186/s12872-022-02932-y

Following publication of the original article [1], In Fig. 3 of this article “The last bracket of "Lp(a) increased ()" was supposed to have positive and negative signs”; the figure should have appeared as shown below.

The original article has been corrected.

**Fig. 3:**
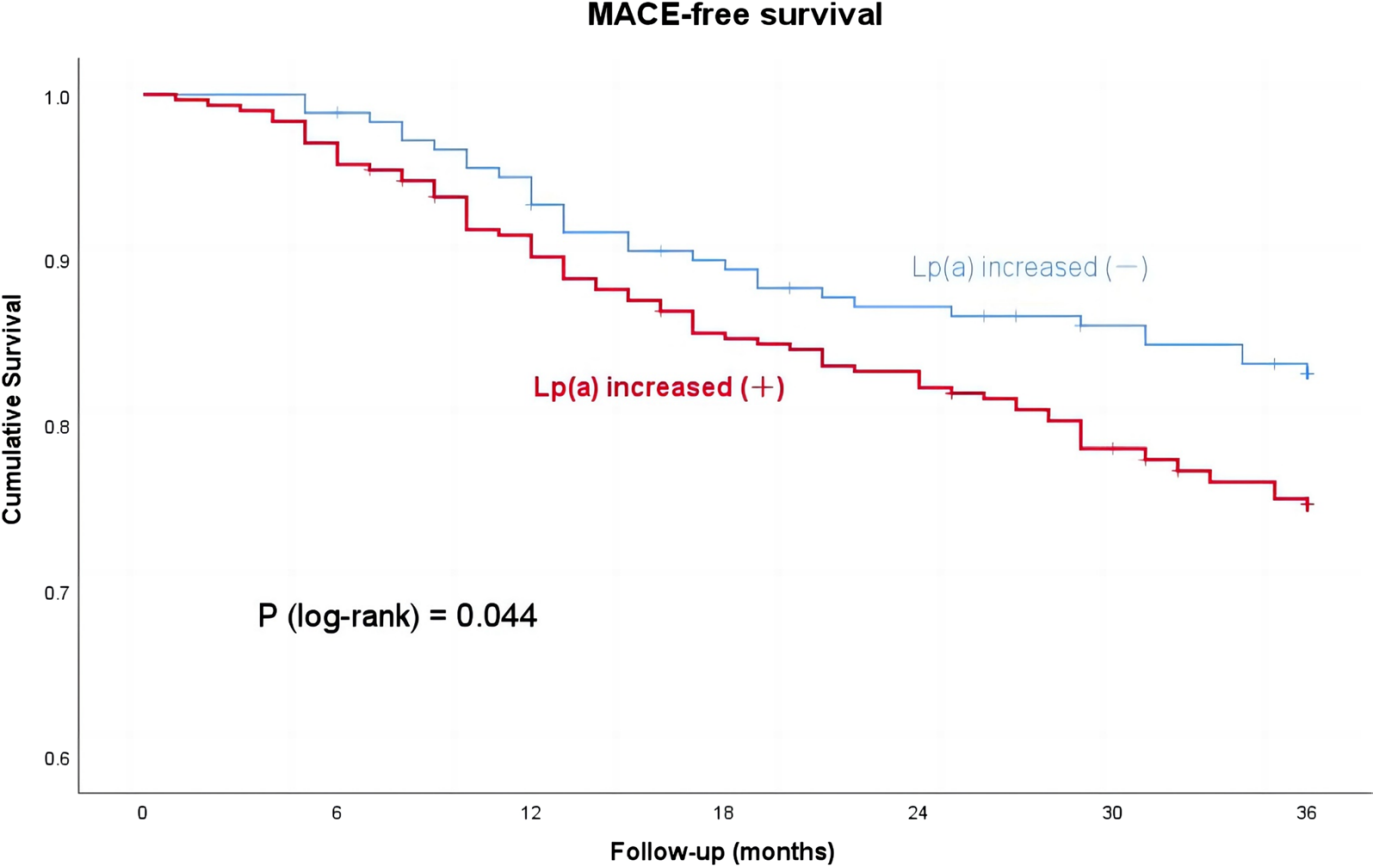
Kaplan–Meier curve model for MACE. *MACE* major adverse cardiovascular events

## References

[1] Zhu L (2022). Effect of an increase in Lp(a) following statin therapy on cardiovascular prognosis in secondary prevention population of coronary artery disease. BMC Cardiovasc Disord.

