# Supplementary material for: Effect of an increase in Lp(a) following statin therapy on cardiovascular prognosis in secondary prevention population of coronary artery disease
Source: BMC Cardiovasc Disord. 2022 Nov 8;22:474. doi: 10.1186/s12872-022-02932-y (PMC9644478; doi:10.1186/s12872-022-02932-y)
Supplement: Supplementary file 1 — Additional file 1: Table S1. Statins used in study subjects. Table S2. Endpoint events for study subjects. Table S3. Univariate COX analysis of risk factors for MACE. [file 12872_2022_2932_MOESM1_ESM.docx]

Table S1. Statins used in study subjects

|  | Lp(a) increased (+)（n=307) | Lp(a) increased (-) (n=181) |
| --- | --- | --- |
| **High-intensity statins[cases (%)]** | 3 (0.98) | 4 (2.21) |
| Atorvastatin 40mg [cases (%)] | 2 (0.65) | 3 (1.66) |
| Rosuvastatin 20mg [cases (%)] | 1 (0.33) | 1 (0.55) |
| **Moderate-intensity statins[cases (%)]** | 304 (99.02) | 177 (97.79) |
| Atorvastatin 20mg [cases (%)] | 204 (66.45) | 134 (74.04) |
| Rosuvastatin 10mg [cases (%)] | 96 (31.27) | 42 (23.20) |
| Atorvastatin 10mg [cases (%)] | 1 (0.32) | 1 (0.55) |
| Rosuvastatin 5mg [cases (%)] | 1 (0.33) | 0 |
| Simvastatin 20mg [cases (%)] | 1 (0.32) | 0 |
| Pravastatin 40mg [cases (%)] | 1 (0.33) | 0 |
| **Low-intensity statins [cases (%)]** | 0 | 0 |

| Endpoint events | Lp(a) increased (+)（n=307) | Lp(a) increased (-) (n=181) | Total (n=488) |
| --- | --- | --- | --- |
| MACE [cases (%)] | 75 (24.43) | 30 (16.57) | 105 (21.52) |
| cardiovascular death [cases (%)] | 7 (2.28) | 3 (1.66) | 10 (2.05) |
| myocardial infarction [cases (%)] | 11 (3.58) | 2 (1.10) | 13 (2.66) |
| ischemic stroke [cases (%)] | 5 (1.63) | 4 (2.21) | 9 (1.84) |
| hospitalizations related to UA [cases (%)] | 27 (8.79) | 14 (7.73) | 41 (8.40) |
| unplanned coronary revascularization [cases (%)] | 25 (8.14) | 7 (3.87) | 32 (6.56) |

Table S2. Endpoint events for study subjects

MACE = major adverse cardiovascular events ; UA = unstable angina

Table S3. Univariate COX analysis of risk factors for MACE

|  | HR | 95%CI | *p* |
| --- | --- | --- | --- |
| Age | 1.024 | 1.003-1.046 | 0.023 |
| gender | 1.115 | 0.745-1.669 | 0.596 |
| BMI | 0.989 | 0.930-1.052 | 0.718 |
| Hypertension history | 1.039 | 0.667-1.618 | 0.867 |
| Diabetes history | 1.731 | 1.178-2.544 | 0.005 |
| Smoking history | 1.028 | 0.700-1.508 | 0.890 |
| Family history | 1.162 | 0.605-2.229 | 0.652 |
| Creatinine | 1.008 | 0.998-1.018 | 0.101 |
| hs-CRP | 1.025 | 1.008-1.042 | 0.003 |
| LDL-C | 1.472 | 1.067-2.029 | 0.018 |
| Lp(a) | 1.008 | 1.002-1.014 | 0.008 |
| HDL-C | 1.119 | 0.975-1.284 | 0.108 |
| TG | 0.961 | 0.745-1.238 | 0.756 |
| Left main lesions | 1.388 | 0.777-2.481 | 0.268 |
| Total coronary occlusion | 1.465 | 0.945-2.269 | 0.088 |
| Number of diseased vessels | 1.686 | 0.958-2.969 | 0.070 |
| Number of coronary stents | 1.069 | 0.955-1.195 | 0.246 |
| Statin | — | — | — |
| Ezetimibe | 0.578 | 0.213-1.569 | 0.282 |
| β blockers | 0.956 | 0.582-1.571 | 0.859 |
| ACEI/ARB | 1.326 | 0.835-2.106 | 0.232 |
| Aspirin | — | — | — |
| Clopidogrel | 0.869 | 0.558-1.354 | 0.536 |
| Ticagrelor | 1.150 | 0.739-1.792 | 0.536 |

BMI = body mass index; hs-CRP =high sensitivity C-reactive protein; LDL-C = low density lipoprotein cholesterol; HDL-C = high density lipoprotein cholesterol; TG = triglycerides; Lp(a) = Lipoprotein (a); ACEI = angiotensin converting enzyme inhibitor; ARB = angiotensin-receptor blocker.
